# Epidemic zoster and AIDS.

**DOI:** 10.3201/eid0204.960417

**Published:** 1996

**Authors:** D M Morens, A K Agarwal, S Sarkar, S Panda, R Detels


10. van Ripper III C, Van Ripper SG, Lee Goff M, Laird M. The epizootiology and ecological significance of malaria in Hawaiian land birds. Ecological Monographs 1986;56:127-44.
11. Buisson GPE. Les îles Marquises et les Marquisiens. Annales d'Hygiène et de Médecine Coloniales 1903;6:535-59.
12. Halstead SB. Pathogenesis of Dengue: challenge to molecular biology. Science 1988;239:476-81.
13. Turner TB. Studies on the relationship between yaws and syphilis. American Journal of Hygiene 1937;25:477-506.
14. Garenne M, Aaby P. J Infect Dis 1990;161:1088.
15. Rollin L. Moeurs et coutumes des anciens Maoris des îles Marquises. Papeete: Stepolde, 1974.
16. Hanson A. Rapa, une île polynésienne hier et aujourd'hui. Paris: Publications de la Société des Océanistes, 1973.
17. Vallaux F. Mangareva et les Gambier. Papeete: Etablissement Territorial d'Achats Groupés, 1994.

### Epidemic Zoster and AIDS

**To the Editor:** Zoster (exogenously reactivated varicella-zoster virus infection) may seem an unlikely candidate for emergence and epidemicity. A recent report, however, describes a zoster outbreak associated with epidemic HIV in injecting drug users in Manipur State, India (1). In addition to underscoring the variety of ways in which "old" diseases may reemerge under complex bio-ecologic conditions, this outbreak may also have implications for anticipating and diagnosing HIV infections and AIDS in developing countries. The Manipur outbreak was associated with a doubling of zoster frequency above background levels, with increased occurrence most notable in males 12-44 years old, who also had the highest HIV prevalence. In a separately studied group of 120 injecting drug users, 20 developed zoster and all were found to be HIV positive (1), a correlation substantially greater than for such other clinical predicters of HIV infection as persistent lymphadenopathy, weight loss, or recurrent dermatoses. Increased zoster occurrence associated with HIV transmission has also been seen in Ho Chi Minh City, Vietnam, and in other Southeast Asian countries, particularly in injecting drug using populations (unpublished). Zoster as a sentinel indicator of community HIV transmission is also suggested by reports from Africa (2).

For over 150 years, it was believed that zoster occurred in local epidemics (3,4). By the 1950s, however, it was generally agreed that zoster represented reactivation of latent gangliar varicella virus either sporadically, or in response to immunosuppression or trauma. Epidemics of "endogenous" immunosuppression, such as those associated with epidemic HIV infection, might thus be expected to produce outbreaks of zoster, as seems to have occurred in Manipur and Vietnam. In the Indian outbreak traumatic zoster seemed unlikely: truncal and facial dermatomes predominated, rather than dermatomes corresponding to drug injection sites (usually the hands or legs). Recognition of zoster outbreaks may be important in developing countries where HIV diagnosis is limited, CD4 cell counts are unavailable, and diagnosis of AIDS is delayed. Zoster is not currently accepted as an AIDS-defining condition (5), and the extent to which it may reflect immune collapse or predict HIV disease progression is uncertain. Nevertheless, greater awareness of zoster as a sentinel indicator of community HIV transmission may be of help not only in clinical diagnosis, but also in public health efforts to recognize epidemic HIV occurrence.

**D. M. Morens,* A.K. Agarwal,† S. Sarkar,† S. Panda,† and R. Detels‡**
*University of Hawaii School of Medicine, Honolulu, Hawaii; ICMR Unit for Research on AIDS in North-eastern States of India, Calcutta, India†; and University of California at Los Angeles, Los Angeles, California‡

### References

1. Panda S, Sarkar S, Mandal BK, Singh TBK, Lokendra Singh K, Mitra DK, et al. Epidemic of herpes zoster following HIV epidemic in Manipur, India. J Infect 1994;28:167-73.
2. Tyndall MW, Nasio J, Agoki E, Malisa W, Ronald AR, Ndinya-Achola JO, Plummer A. Herpes zoster as the initial presentation of human immunodeficiency virus type 1 infection in Kenya. Clin Infect Dis 1995;21:1035-7.
3. Simon L. *Questions sur diverses branches des sciences médicales. Thèse 202.* Paris: Rignoux, 1840.
4. Kaposi M. Bemerkungen über die jüngste Zoster-Epidemie und zur Aetiologie des Zoster. Wien Med Wochenschr 1889;25:961-4, 26:1001-4.

5. World Health Organization. Interim proposal for a WHO staging system for HIV infection and diseases. Wkly Epidemiol Rec 1990;65:221-4.

### Ancient Egypt and Today: Enough Scourges to Go Around

**To the Editor:** In a recent letter (1), Ablin conjectures that translation of the hieroglyphic symbol for *AAA* in many ancient Egyptian papyri (Ebers, Berlin, Hearst, London, and Kahum), may be suggesting the existence of human immunodeficiency virus (HIV) or its prototype during the time of the pharaohs. While hieroglyphic interpretations remain challenging, the symbol cited in his letter has most commonly been translated as hematuria (2-4) and has most often been related to schistosomiasis haematobia. This infection, caused by the helminth *Schistosoma haematobium*, has been shown to have occurred in Egypt from early pharaonic times (3200 B.C.), by the demonstration of schistosome eggs (5) and circulating schistosome antigens (6,7) in mummies. Remedies for hematuria were recorded in papyri from many centuries (9 in Hearst, 11 in Berlin, 20 in Ebers), perhaps implying that the condition was serious and widespread. In giving one of the remedies in the Ebers papyrus (circa 1500 B.C.), the text actually mentions worms in the body (although it seems to state that the worms are caused by *AAA* disease, perhaps inverting the true order of causality). In the Hearst papyrus one of the remedies cited for hematuria is antimony disulfide. Until only 25 years ago, antimonial compounds were the most effective drugs for schistosomiasis chemotherapy.

It seems likely that, over a period of many centuries in ancient Egypt, *AAA* disease was a widespread condition of sufficient severity to require medical attention. I concur with many others in proposing that the translation of *AAA* disease is hematuria, and that the relationship drawn between *AAA* and worms in the body, antimonial-based remedies, and the knowledge that *S. haematobium* infections were widely present at that time provide strong evidence that *AAA* disease refers to schistosomiasis haematobia.

Schistosomiasis is still with us. In fact, through dispersions of both human populations and specific fresh-water snails (the intermediate hosts for schistosomes), this disease now infects some 200 million persons and is responsible for an estimated 800,000 deaths per year (8). While clearly ancient, schistosomiasis can emerge as a new infectious disease in a given location under certain man-made changes in environmental conditions and economic- or war-related migrations of people. For example, in the Senegal River basin, estuarine dams, irrigation systems, and an influx of people to work irrigation-intense crops led, over a period of only 3 years, to an increased prevalence of *S. mansoni* infection from 0% to >95% of the population of >50,000 (9). Even in modern-day Egypt, such interventions as the Aswan High Dam have significantly altered patterns of schistosomiasis (2,10). The Ministry of Health and Population of Egypt and the U.S. Agency for International Development are addressing this ancient scourge through the Schistosomiasis Research Project, a national schistosomiasis research and control program that attacks the disease with available tools, while it presses forward with research on much needed new tools, such as vaccines.

**Daniel G. Colley**
Centers for Disease Control and Prevention, Atlanta, Georgia, USA

### References

1. Ablin RJ. AIDS: déjà vu in ancient Egypt? Emerging Infectious Diseases 1996;2:242.
2. Abdel-Wahab MF. Schistosomiasis in Egypt. Boca Raton: CRC Press, Inc., 1982.
3. Farooq M. Historical development. In: Ansari N, editor. Epidemiology and control of schistosomiasis (bilharziasis). Baltimore: University Park Press, 1973:1-16.
4. Kolata G. Avoiding the schistosome's tricks. Science 1985;227:285-7.
5. Ruffer M. Note on the presence of *Bilharzia haematobium* in Egyptian mummies of the twentieth dynasty (1250-1000 B.C.). Br Med J 1910;1:16.
6. Deelder AM, Miller RL, de Jonge N, Krijger FW. Detection of antigen in mummies. Lancet 1990;335:724-5.

